# The reductive decyanation reaction: an overview and recent developments

**DOI:** 10.3762/bjoc.13.30

**Published:** 2017-02-13

**Authors:** Jean-Marc R Mattalia

**Affiliations:** 1 Aix-Marseille Univ, CNRS, Centrale Marseille, iSm2, Marseille, France

**Keywords:** α-aminonitrile, decyanation, electron donor, hydride, malononitrile, transition metal catalysis

## Abstract

This review presents an overview of the reductive decyanation reaction with a special interest for recent developments. This transformation allows synthetic chemists to take advantages of the nitrile functional group before its removal. Mechanistic details and applications to organic synthesis are provided.

## Introduction

Many strategies in organic synthesis involve the removal of a beneficial functional group. The electron-withdrawing properties of the nitrile functional group appear beneficial in a variety of reactions [1–2]. This group coordinates metal complexes and can be used as a directing group for C–H bond activation reactions catalyzed by transition metals [3–6]. The α-deprotonation of alkylnitriles generates active α-cyano carbanion nucleophiles. Recent investigations have resulted in different modes of alkylnitrile activations and in the development of new catalytic cyanoalkylation methodologies [7]. While Fleming and Zhang first focused on the removal of the cyano group from cyclic substrates [8], in 2006 we published a review reporting various methods allowing the reductive decyanation reaction that transforms organic nitriles into the parent alkane [9]. Even if chemical procedures previously described are still of relevance in organic synthesis, it is noteworthy that new methods have now emerged with the aim to develop mild reaction conditions that allow reduction of a wide scope of substrates and tolerate a variety of functional groups. This review attempts to be complementary to the paper published in 2006 and proposes an overview of the reductive decyanation reaction that focuses on more modern methods.

## Review

### Alkali-metal-promoted decyanation

**Scheme 1:**
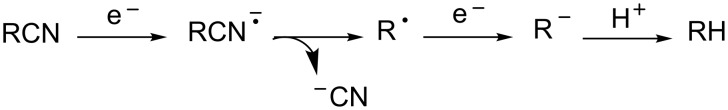
Mechanism for the reduction under metal dissolving conditions.

Since the article by Arapakos in 1967 [10], decyanations using alkali metal dissolving conditions, typically Li or Na/NH_3_, are widely used in organic synthesis [11–15]. The mechanism proposed involves an electron transfer with formation of a radical anion intermediate, and then a cyanide anion is eliminated with concomitant formation of a radical. The latter is then reduced to a carbanion which can be in situ protonated by ammonia or, depending on the conditions used, another proton source (Scheme 1).

**Scheme 2:**
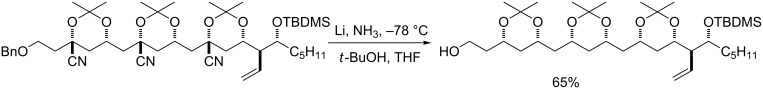
Example of decyanation in metal dissolving conditions coupled with deprotection [30]. TBDMS = *tert*-butyldimethylsilyl.

Using Na/NH_3_ or Li/EtNH_2_ solutions, Arapakos obtained the best yields for the decyanation of phenyl-substituted acetonitriles, tertiary alkyl, and aromatic nitriles. However, primary and secondary nitriles also led to the reduction to the amine [10,16]. This drawback can be overcome using K/HMPA/*t*-BuOH [17–18] or K/dicyclohexano-18-crown-6/toluene [19–20]. In the latter case, the toluene radical anion is believed to be the reactive species. LiDBB (lithium di-*tert*-butylbiphenylide) and Li naphthalenide are also common electron donors [15,21–22]. Because of the mechanism described in Scheme 1, the nature of the medium and the substrate strongly influence the course of the reaction. Then, in the absence of a proton source, the organolithium intermediate can cyclize or react with an electrophile giving the expected coupling products [23–27]. Metal dissolving conditions allow the reduction of various other functional groups [28]. Rychnovsky took advantage of this reactivity and achieved reductive decyanations with concomitant Birch reduction or benzyl ether cleavage [29–31]. An example related to the synthesis of polyene macrolides is described in Scheme 2.

**Scheme 3:**
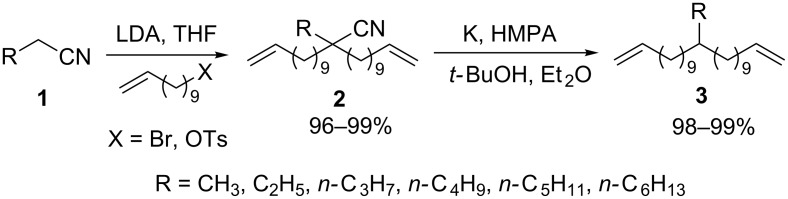
Preparation of α,ω-dienes [18,33].

Rojas et al. proposed a convenient two-step pathway for the preparation of alkyl α,ω-dienes **3**. These dienes are well-known precursors in ring-closing metathesis (RCM) and acyclic diene metathesis (ADMET) chemistry [32]. They first reported the quantitative α-alkylation of primary nitriles **1** [33]. In a second part of their work, they developed adequate conditions to carry out the decyanation reaction without olefin isomerization [18]. They explored several methods for the preparation of 12-butyltricosa-1,22-diene **3** (R = *n-*C_4_H_9_). The reaction was carried out in a slurry of K/Al_2_O_3_ in hexane, hexane/toluene (1:1) and toluene giving 20%, 63% and 75% of olefin isomerization (from NMR and GC), respectively, for each solvent [34]. This isomerization was attributed to the translocation of the tertiary radical intermediate to a more stable allyl radical leading to the double bond migration. This rearrangement was avoided using K/Ph_3_CH in hexane/ether (**3** R = *n-*C_4_H_9_, 41% yield) or K/HMPA/*t*-BuOH in ether (**3** R = *n-*C_4_H_9_, 99% yield). The latter optimized conditions allow the decyanation of alkylcyano α,ω-dienes **2** in quantitative yields with no detection of olefin isomerization (Scheme 3).

**Scheme 4:**
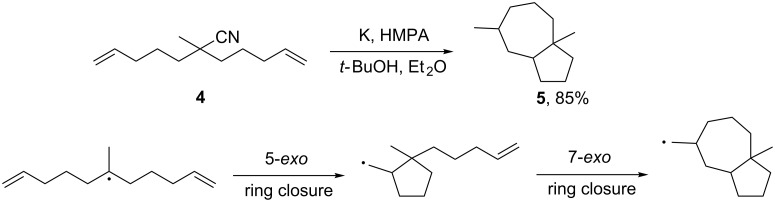
Cyclization reaction using a radical probe [18].

Radical intermediates were trapped using the radical probe **4** (Scheme 4). Replacing *t*-BuOH with *t*-BuOD in Scheme 3 (R = CH_3_ case), yielded the decyanation product **3** with 92% deuterium incorporation. With respect to the mechanism described in Scheme 1, the authors suggest that *t*-BuOH (or Ph_3_CH) could act as H-atom donor that quenches the radical intermediate. However, this interpretation is opened to discussion because olefin functionalities also can undergo isomerization via anionic intermediates [35] and radicals usually abstract hydrogen atoms preferentially from the alkyl groups of *t*-BuOH [36–38]. Therefore *t*-BuOH could act as a proton donor and so prevent the olefin isomerization.

**Scheme 5:**
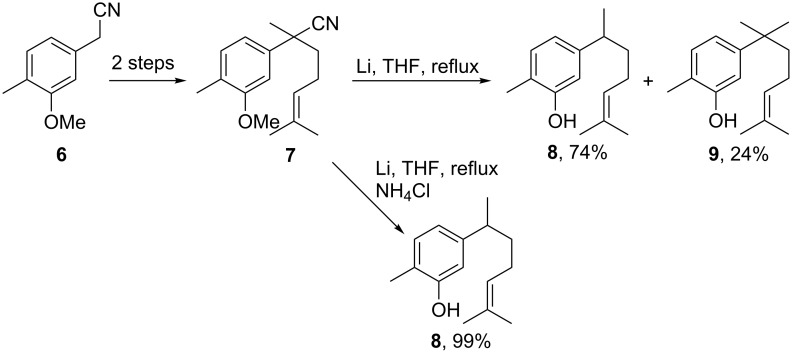
Synthesis of (±)-xanthorrhizol (**8**) [39].

Alkali metals can also be used in suspension. As mentioned above, highly dispersed potassium over neutral alumina (K/Al_2_O_3_) in hexane is able to effect the reductive cleavage of alkylnitriles [18,34]. Zárraga et al. described an efficient synthesis of (±)-xanthorrhizol (**8**) [39]. The authors prepared the intermediate **7** by dialkylation of **6** and attempted to carry out a one-pot decyanation and demethylation [40] with a suspension of lithium in THF. The target compound **8** was obtained in 74% yield together with 24% of the byproduct **9** (Scheme 5). This compound seems to be formed by a cross-linked ether cleavage of the methoxy group induced by the anion intermediate resulting from the decyanation pathway (Scheme 1). The authors increased the yield to 99% by adding NH_4_Cl as proton source that immediately reacts with the anion before the ether cleavage.

### Aluminium- and borohydrides and the use of sodium hydride

#### Reduction of α-aminonitriles

**Scheme 6:**
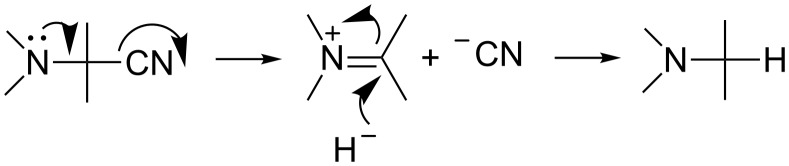
Mechanism for the reduction of α-aminonitriles by hydride donors.

Bifunctional α-aminonitriles exhibit several modes of reactivity. Recent reviews demonstrated the richness of this chemistry and emphasized synthetic applications particularly in heterocyclic chemistry [41–44]. The reductive decyanation of α-aminonitriles under metal dissolving conditions is a common procedure that proceeds through a two-electron-transfer pathway (Scheme 1) [23,44]. In the ionic pathway, the loss of the cyanide ion yields an iminium cation that can be reduced by various hydride donors (Scheme 6). Alternatively, secondary amines could involve an elimination of HCN and reduction of the formed imine.

NaBH_4_ [45–51] or NaBH_3_CN [47,52–59] are widely used hydride donors and, less frequently, BH_3_ [60–61], AgBF_4_/Zn(BH_4_)_2_ [62–64] or LiAlH_4_ [65–66]. These reactions usually require mild conditions but the course of the reaction depends on the ease of formation of the iminium ion [48,67–69]. With the highly reactive LiAlH_4_, diamines can also be formed by reduction of the nitrile moiety [70]. This competition with the decyanation reaction depends on the structure of the α-aminonitrile, stereoelectronic effects and internal strain of the molecule [68].

**Scheme 7:**
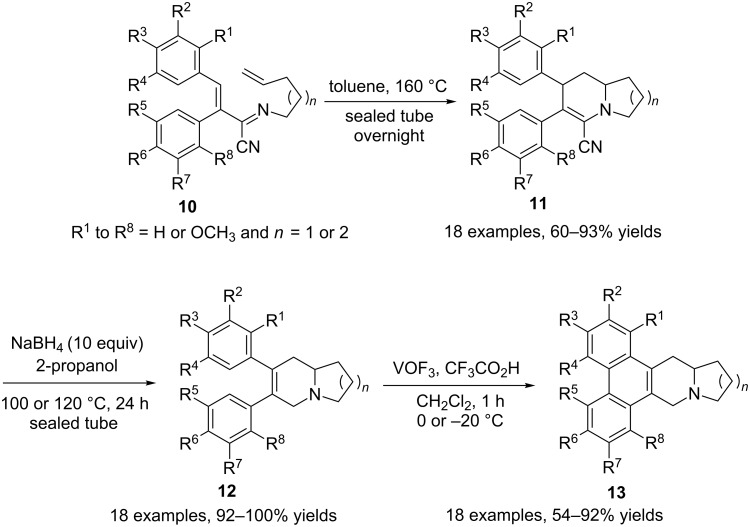
Synthesis of phenanthroindolizidines and phenanthroquinolizidines [71].

Chuang et al. prepared a set of α-aminoacrylonitriles **11** by a cyano-promoted aza-Diels–Alder cycloaddition [71]. The cyano groups were then removed in high yields by treatment with NaBH_4_ in 2-propanol by using both basic and nucleophilic properties of the hydride ion. The proposed mechanism involves a double-bond isomerization to the α-aminonitrile intermediate which is then reduced by the hydride ion in a classical way (Scheme 7). Interestingly, deuterium-labelling experiments indicate that one of the methylene hydrogens of the formed allylamine **12** is derived from the protic solvent and the other comes from the reducing agent. Finally, an oxidative aryl–aryl coupling promoted by vanadium oxytrifluoride (VOF_3_) afforded phenanthroindolizidines (**13**, *n* = 1) and phenanthroquinolizidines (**13**, *n* = 2). Anticancer activities of these 18 compounds were evaluated against three human cancer cell lines.

**Scheme 8:**
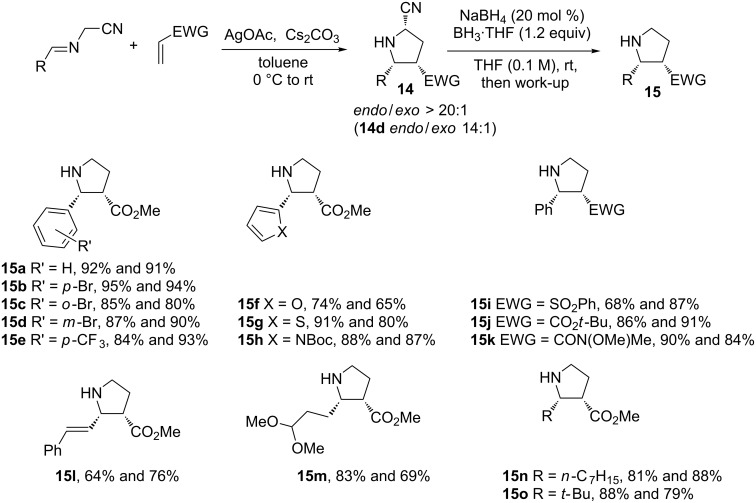
Two-step synthesis of 5-unsubstituted pyrrolidines (25 examples and 1 synthetic application, see below). The two yields refer successively to the cycloaddition (**14**) and decyanation (**15**) steps [74]. Boc = *tert*-butoxycarbonyl. EWG = electron-withdrawing group.

BH_3_·THF containing NaBH_4_ has been used for the reduction of diimines [72–73] and was studied in-depth by Zhang and co-workers in the reductive decyanation reaction. In their work, the cyano group activates the [3 + 2] cycloaddition of azomethine ylides and is then removed to yield 5-unsubstituted pyrrolidines [74]. These substructures appear in several biologically active natural products and drugs [75]. A range of decyanation conditions were screened such as NaBH_4_ in THF or MeOH, NaBH_4_/AgBF_4_ in THF and NaBH_3_CN in MeOH/AcOH. They also explored BH_3_ alone in THF and with varying amounts of NaBH_4_. They found that the addition of a catalytic amount of NaBH_4_ was very efficient for the reductive decyanation reaction. Scheme 8 shows the scope of this two-step transformation. This protocol is successfully used with various electron-deficient substituted phenyl groups (**15b**–**e**) and with heterocycles (**15f–h**). The double-bond of cyanopyrrolidine **14l** is preserved from the hydroboration reaction (**15l**). Olefins bearing bulky ester, sulfone or amide groups afforded good to excellent yields (**15i–k**). A series of aliphatic α-iminonitriles also gave good results (**15n**,**o**). Notably the labile dimethylacetal group tolerates this two-step transformation (**15m**).

**Scheme 9:**
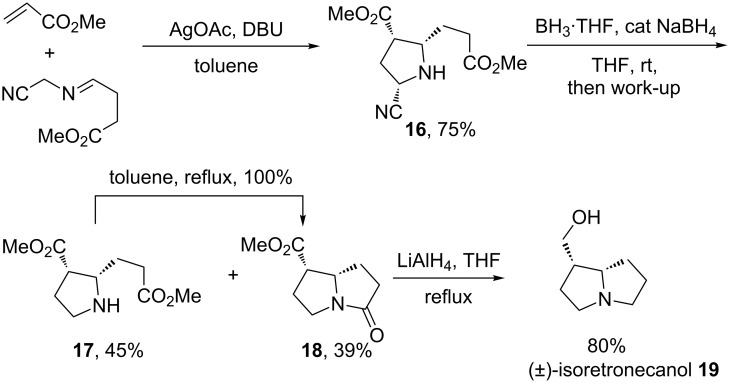
Synthesis of (±)-isoretronecanol **19**. DBU = 1,8-diazabicyclo[5.4.0]undec-7-ene [74].

Using this protocol, the authors described a total synthesis of (±)-isoretronecanol (**19**), a pyrrolizidine alkaloid (Scheme 9). In this case the two-step protocol is followed by a lactamization and the one-pot reduction of ester and lactam groups of **18**.

**Scheme 10:**
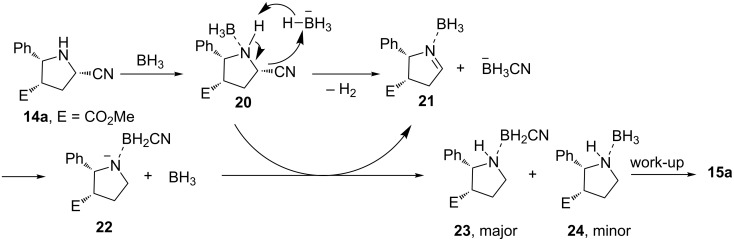
Proposed mechanism with **14a** for the NaBH_4_ induced decyanation reaction (“BH_3_” = BH_3_·THF) [74].

The authors proposed a reductive anionic chain mechanism described in Scheme 10. The exposure of **14a** to BH_3_ generates a borane–amine complex **20** whose fragmentation could be promoted by NaBH_4_. The resulting imine **21** is reduced by BH_3_ with the help of the cyanoborohydride anion. The formed anion **22** abstracts a proton from complex **20** to produce **23** or **24** and regenerate **21** and BH_3_CN^−^. A set of experiments supports this proposal. Notably, borane is the major hydride source for the reduction and **22** (derived from the reaction of **23** and NaH) is efficient for this chain reaction. Stable intermediates **23** and **24** were fully characterized while the unstable complex **20** was only characterized with ^1^H NMR.

#### Reduction of other substrates

The reductive decyanation promoted by aluminium- and borohydrides for substrates other than α-aminonitriles has been described for more specific cases and usually displays moderate yields [76–79]. Recently, the DIBAL-H-induced decyanation of *gem*-dicyanodihydroazulene derivatives was described but only poor yields were reported [80].

**Scheme 11:**
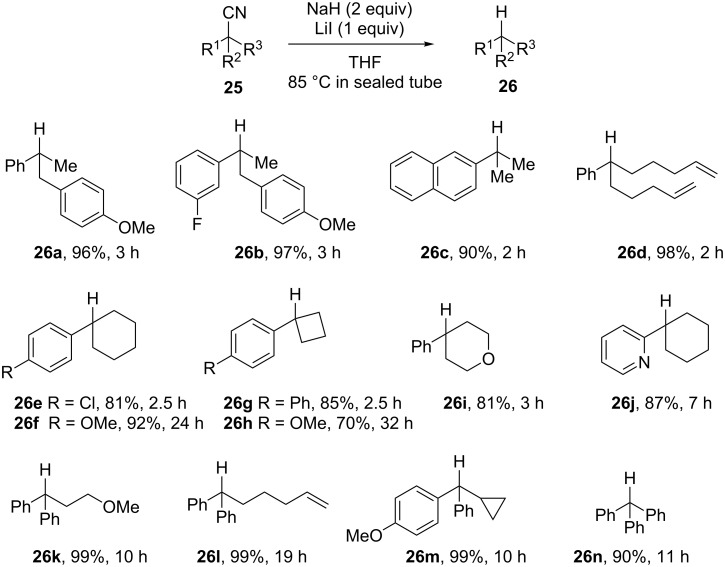
Reductive decyanation by a sodium hydride–iodide composite (26 examples) [81].

Chiba et al. accidently discovered the reductive decyanation of aryl substituted tertiary nitriles (Scheme 11) [81]. The protocol involves NaH in THF in the presence of LiI at 85 °C and appears suitable for the construction of the important 1,1-diarylalkane (**26k**–**m**) and triarylmethane (**26n**) derivatives. Strained cyclobutylarenes (**26g**,**h**) and heterocycles (**26i**,**j**) are prepared in this way and the electron-rich 4-methoxyphenyl group decreases the reaction rate (compare **26e**,**g** with **26f**,**h**). The decyanation of nitriles with the NaH–NaI system gives comparable yields but much longer reaction times are generally required.

The reduction of radical probes (no rearranged products formed from **25d**,**l**,**m**) and deuterium-labelling experiments (no deuterium incorporation using THF-*d*_8_ and quenching with D_2_O) discard the possibility of a single-electron transfer pathway. Other reductions suggest a hydride addition with formation of an iminyl anion intermediate. Particularly, when the reaction of **25f** was quenched after 2.5 h, the corresponding aldehyde was formed in 42% yield together with 37% of the decyanated product **26f**.

**Scheme 12:**
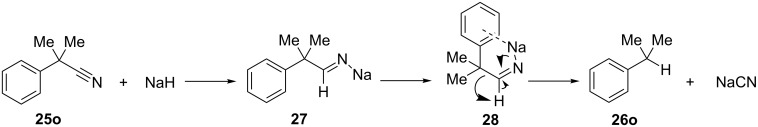
Proposed mechanism for the reduction by NaH [81].

DFT calculations conducted on nitrile **25o** support the hydride addition to the CN triple bond with formation of an iminyl anion intermediate **27**. The latter easily isomerizes to its isomer **28** where a sodium cation*–*π-interaction occurs. The last step involves a C–C bond cleavage and proton transfer with elimination of NaCN (Scheme 12). This proton transfer occurs with retention of configuration as experimentally observed. Indeed, the kinetic profiles show that the decyanation reactions include an induction period (0.5 h and 2 h, respectively, for NaH–LiI and NaH–NaI systems) suggesting the formation of a new inorganic composite. These materials consist of metal iodide interspersed with activated NaH resulting in an unique hydride-donor reactivity [82]. An addition–elimination mechanism has been previously proposed for the LiAlH_4_ promoted decyanation of 2,2-diphenylpropionitrile and related nitriles. In such pathways, the phenyl groups probably favor the C–C bond cleavage by stabilizing the incipient negative charge on the carbon adjacent to the cyano group [76–77].

### Transition-metal-catalyzed reductive decyanation

#### Hydrogenation of α-aminonitriles

**Scheme 13:**
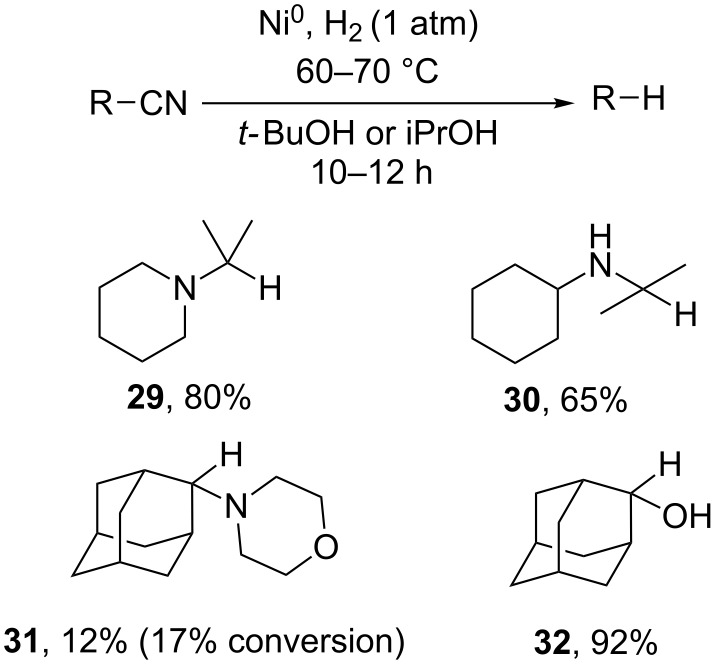
Reductive decyanation catalyzed by nickel nanoparticles. Yields are given in weight % from GC–MS data [85].

The decyanation of α-aminonitriles with hydrogen present in an excess of Raney nickel was described by Husson and co-workers on oxazolidine derivatives [83–84]. The authors logically proposed that the decyanation occurred via the reduction of an iminium ion intermediate. The hydrogenation of α-aminonitriles catalyzed by nickel nanoparticles results in reductive decyanation and yields **29–31**. The colloid solution of nickel is prepared in situ via reduction of anhydrous NiCl_2_ with NaBH_4_ in *t*-BuOH, iPrOH or *n*-butanol, the reactions are then performed upon bubbling of hydrogen at atmospheric pressure through the reaction mixture (Scheme 13). The reductive decyanation of 2-hydroxyadamantane-2-carbonitrile is successful by this method and yields alcohol **32** [85].

**Scheme 14:**
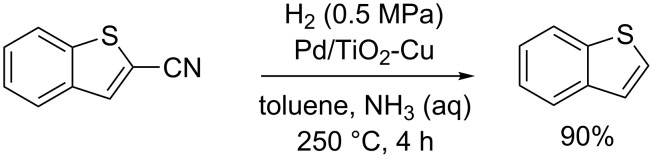
Decyanation of 2-cyanobenzo[*b*]thiophene [87].

For less reactive nitriles, the C–CN bond cleavage usually requires hydrogen under pressure and high temperatures [86]. Tao et al. reported the decyanation of 2-cyanobenzo[*b*]thiophene using hydrogen and Pd, Pt/TiO_2_ or Pd/TiO_2_-Cu as catalyst at 200–300 °C with yields varying from 79 to 90% (Scheme 14) [87].

Opatz et al. developed the enantioselective syntheses of various alkaloids using the rhodium catalyst developed by Noyori [88] for the asymmetric transfer hydrogenation of imines. Interestingly, imines are formed from unstable α-aminonitrile intermediates which spontaneously eliminate HCN [89–91].

#### Iron-catalyzed reductive decyanation

**Scheme 15:**
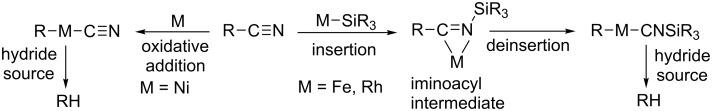
Simplified pathways involved in transition-metal-promoted reductive decyanations [93,95].

In 1982, Yamamoto et al. disclosed the C–CN bond cleavage promoted by an electron-rich cobalt complex [92]. Since that time, the activation of inert C–CN bonds by transition metals has been widely investigated. Improvements towards mild and green conditions with a large substrate scope have been achieved in many reactions including decyanation. Several reviews account for the richness of this chemistry [93–99]. Two major pathways for the C–CN bond activation have emerged (Scheme 15). One is the oxidative addition of the C–CN bond to a low-valent metal center (Ni case). The other pathway involves a silylmetal-assisted carbon cyano bond cleavage through an iminoacyl intermediate (Rh, Fe cases). In both pathways, the combination with a reducing agent gives a catalytic reductive system for the removal of the cyano group.

**Scheme 16:**
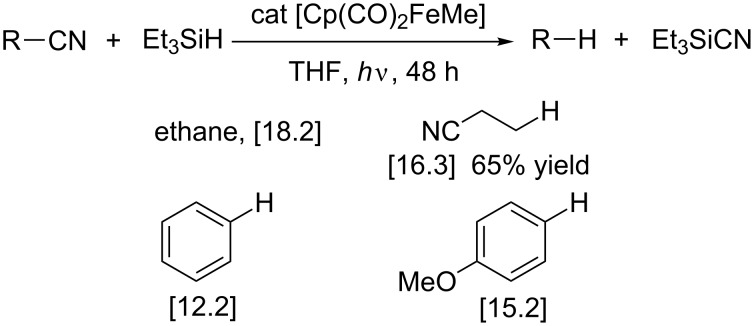
Fe-catalyzed reductive decyanation. Numbers in square brackets represent turnover numbers. The TONs were determined by GC and based on the amount of Et_3_SiCN produced (10 examples with TON > 4) [100]. Cp = cyclopentadienyl.

The discussion below on transition-metal-catalyzed reactions focuses on selected examples that show the wide scope of reduced substrates including alkyl cyanides, challenging substrates due to their propensity to undergo β-hydride elimination from alkylmetal intermediates. Nakazawa et al. reported the photoinduced C–CN bond cleavage catalyzed by iron complexes of a few primary alkyl cyanides and aryl cyanides in the presence of Et_3_SiH [100–101]. Decyanated species were formed together with silyl cyanide. Both aliphatic and aromatic nitriles were successfully reduced (Scheme 16) but an electron-withdrawing, bulky or coordinating substituent on the C atom linked to the cyano group disfavors the reductive decyanation.

#### Rhodium-catalyzed reductive decyanation

**Scheme 17:**
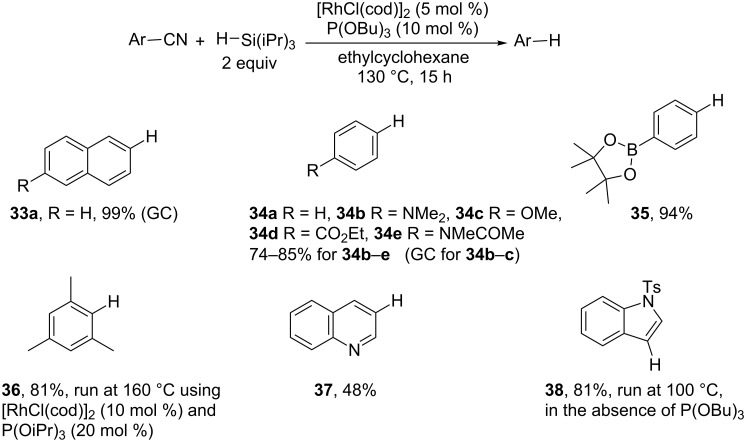
Rh-catalyzed reductive decyanation of aryl nitriles (18 examples, 2 synthetic applications) [103].

**Scheme 18:**
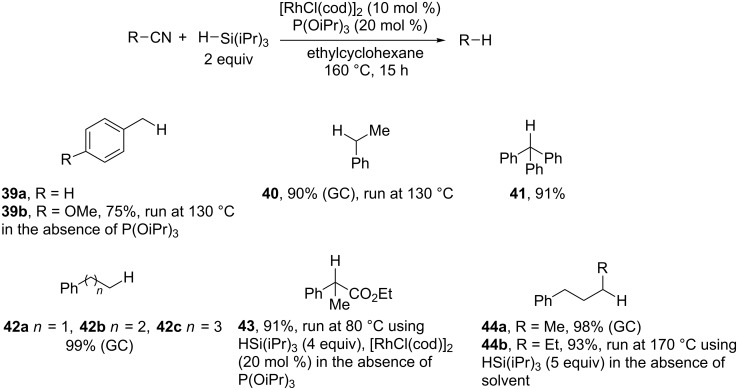
Rh-catalyzed reductive decyanation of aliphatic nitriles (15 examples, one synthetic application) [103].

Chatani and co-workers have investigated the rhodium-catalyzed carbon–cyano bond cleavage reactions using organosilicon reagents [94]. They reported a rhodium-catalyzed reductive decyanation with a hydrosilane as reducing agent. They selected [RhCl(cod)]_2_ (cod = 1,5-cyclooctadiene) as catalyst and found that the use of triisopropylsilane and P(OBu)_3_ or P(OiPr)_3_ as ligands led to the best results [102–103]. Aryl, heteroaryl (Scheme 17), benzyl and alkyl cyanides (Scheme 18) are applicable to this decyanation reaction.

The use of hydrosilane as a mild reducing agent allows a high functional group tolerance (**34b–e**, **35**, **39b**, **43**) and sterically hindered cyano groups are successfully removed (**36**, **41**, **43**). Reactions of benzyl cyanides proceed smoothly even in the absence of the phosphite ligands (**39b**, **43**). Interestingly, an ester group in the α-position increases the reactivity (**43**). The reaction works well with simple primary and secondary alkyl cyanides but requires a higher reaction temperature. Aliphatic nitriles containing β-hydrogen atoms are successfully reduced (**40**, **42a–c**, **43**, **44a**). Even if alkenes are not formed, the authors show the occurrence of a β-hydride elimination/hydrometalation sequence. However, the reduction of the more hindered substrate **44b** requires a larger excess of hydrosilane to prevent the formation of alkene. Finally, unactivated tertiary alkyl cyanides lead to a complex mixture.

#### Nickel-catalyzed reductive decyanation

**Scheme 19:**
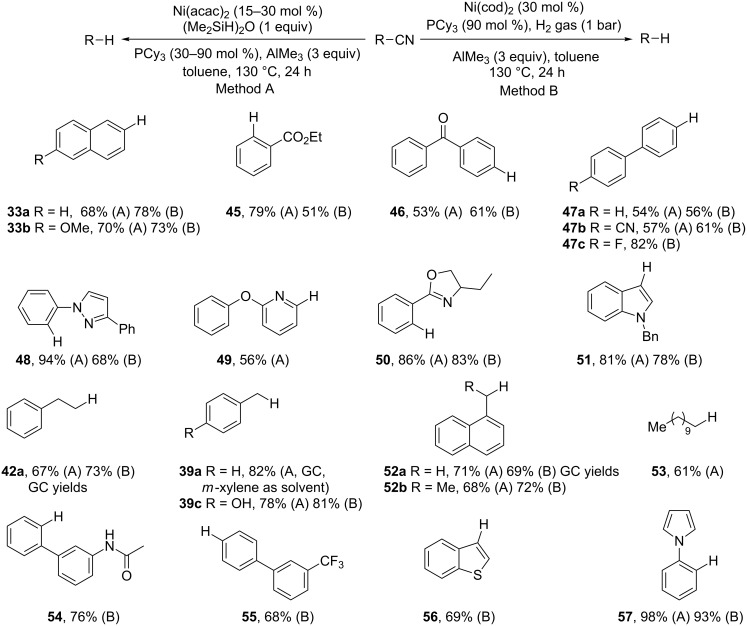
Ni-catalyzed reductive decyanation (method A: 28 examples and 2 synthetic applications; method B: 31 examples and 5 synthetic applications) [104–105].

Maiti et al. established that the Ni(acac)_2_ complex (acac = acetylacetonate) of PCy_3_ (Cy, cyclohexyl) in combination with TMDS (tetramethyldisiloxane) as hydride source can catalyze the reductive decyanation efficiently (method A, Scheme 19) [104]. The use of AlMe_3_ appeared beneficial by facilitating the oxidative addition of the C–CN bond to Ni. Soon after, the same team developed the use of cheaper, greener and milder hydrogen gas as the hydride source. They found that catalytic Ni(cod)_2_ in combination with PCy_3_ and AlMe_3_ under 1 bar pressure of H_2_ gas was the protocol of choice (method B) [105]. As well as the activation of the C–CN bond, AlMe_3_ helps to remove and consume the HCN gas produced under the reaction conditions. These protocols were successfully applied to a wide range of nitriles containing various other functional groups (Scheme 19). The ether bonds (**33b**, **49**), ester (**45**), hydroxy (**39c**) and keto groups (**46**) are tolerated. With method B, aryl cyanides with amide (**54**), trifluoromethyl (**55**) and fluoro groups (**47c**) are reduced in attractive yields. For substrates bearing dicyano groups, monodecyanation is the major pathway (**47b**) and decyanations are efficient with sterically demanding *ortho*-substituents (**54**, **57**) or with *ortho*-directing groups (**45**, **48**, **50**). Aliphatic nitriles underwent decyanation without β-hydride elimination (**42a**, **52b**, **53**).

**Scheme 20:**
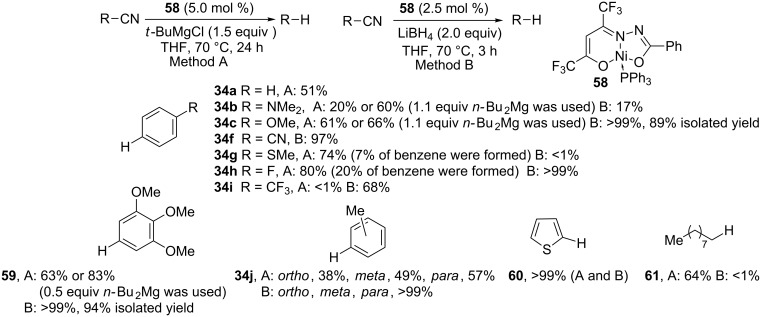
Reductive decyanation catalyzed by the nickel complex **58** (method A, 14 examples, yield ≥ 20% and 1 synthetic application; method B, 12 examples, yield ≥ 17% and 1 synthetic application). Yields determined by GC–MS and ^1^H NMR spectroscopy [106–107].

Enthaler et al. reported the application of nickel complex **58** as precatalyst and *tert*-butylmagnesium chloride as reducing agent (method A) in the decyanation of alkyl and aryl cyanides [106]. In the same year, to easily generate nickel hydride complexes, the authors explored the possibility to apply more reactive hydride donors such as LiBH_4_ (method B) [107–108]. Finally, after investigating the reaction conditions and a precatalyst screening, the conditions described in Scheme 20 were selected. With method B, lower catalyst loading and shorter reaction times are necessary. With method A, benzonitrile yields 51% of benzene (**34a**) and an increase in yield is obtained with the methoxy and methyl groups (**34c**, **59**, **34j ***para*). For some substrates, the replacement of *t-*BuMgCl by *n-*Bu_2_Mg improves the yield (**34b**,**c**, **59**). With method B, excellent yields are obtained for methoxy (**34c**, **59**) and methyl substituted aryl cyanides **34j** while with method A, changing the position of the methyl substituent from the *para* to the *ortho* or *meta* positions decreases the yield in toluene. Dimethylamino and thioether groups display a lower yield with method B (**34b**,**g**) contrary to the trifluoromethyl group (**34i**). With *p*-fluorobenzonitrile the decyanation is observed with both methods (**34h**) but, with the bromo counterpart, dehalogenation also occurs. 2-Cyanothiophene results in quantitative yield of thiophene **60** with both methods but cyanopyridines are not converted, probably due to the coordination abilities of the pyridine. Finally, decanenitrile is decyanated with method A (**61**) but aliphatic nitriles are not converted to the desired product with method B.

**Scheme 21:**
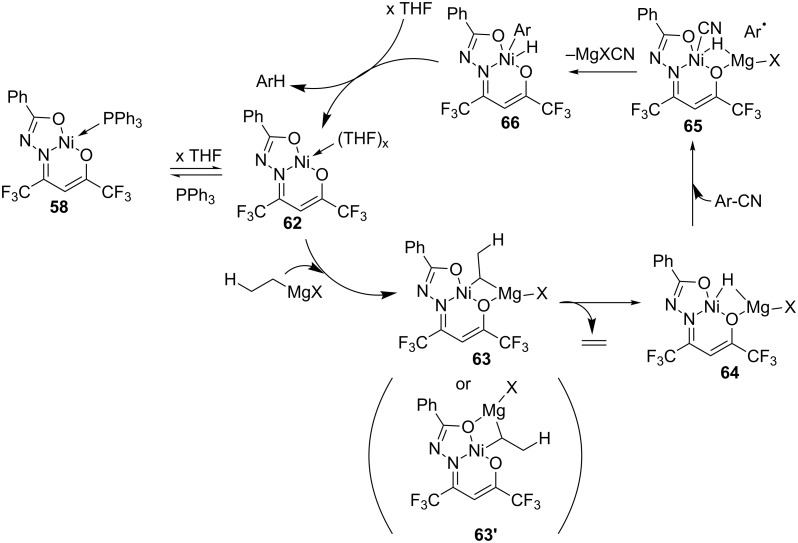
Proposed catalytic cycle for the nickel complex **58** catalyzed decyanation (method A). Only the cycle for **63** is shown [106].

Trapping of radicals with method A led the authors to propose the electron transfer mechanism described in Scheme 21. The complex **62** reacts with the Grignard reagent to form the nickel-magnesium hydride intermediate **64** via a β-hydride elimination from **63**. A single-electron transfer (SET) to the nitrile oxidizes the complex at the metal center into **65** and generates an aryl radical. The electron can also be located in the ligand (non-innocent ligand, not represented in Scheme 21). Then, elimination of MgXCN and radical recombination with the nickel species produce **66**. Finally, an elimination of Ar-H closes the catalytic cycle. A similar mechanism was proposed with method B.

### Radical reactions

This part is related to radical reactions not involving alkali metals and focuses on the reduction of malononitriles and cyanoacetates (α-cyanoesters). These compounds are particularly versatile reagents in organic synthesis including multicomponent reactions [109–113]. However, like decarboxylation for related malonic ester or acetoacetates, an easy access to the removal of the cyano group should encourage future developments using these compounds.

#### Bu_3_SnH and *N*-heterocyclic carbene boranes

**Scheme 22:**
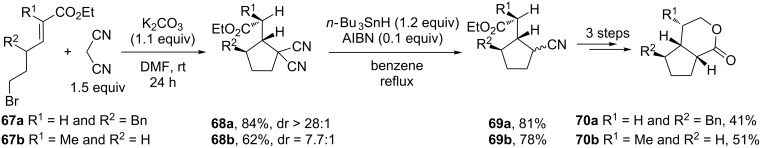
Synthesis of bicyclic lactones [119–120].

The reductive decyanation of malononitriles to mononitriles using tributyltin hydride/AIBN in benzene was unexpectedly discovered by Curran and Seong [114]. Later, they made a full study and successfully reduced to mononitriles a variety of mono- and dialkylated malononitriles but under these conditions, the reduction of cyanoacetates failed [115]. Synthetic applications of this methodology were later described [116–118]. Chiba et al. have developed a concise and stereoselective methodology for the preparation of highly substituted carbocycles [119–120]. An example in described in Scheme 22. The 5-membered ring is formed via a K_2_CO_3_ mediated S_N_2 – conjugate addition sequence between malononitrile and 6-bromo-2-hexenoate derivatives **67a,b**. Dicyanocyclopentanes **68a**,**b** are treated with tributyltin hydride/AIBN giving the monodecyanated products **69a**,**b**. Bicyclic lactones **70a**,**b** are then obtained in 3 steps in 41% and 51% yields, respectively, from **69a**,**b**.

**Scheme 23:**
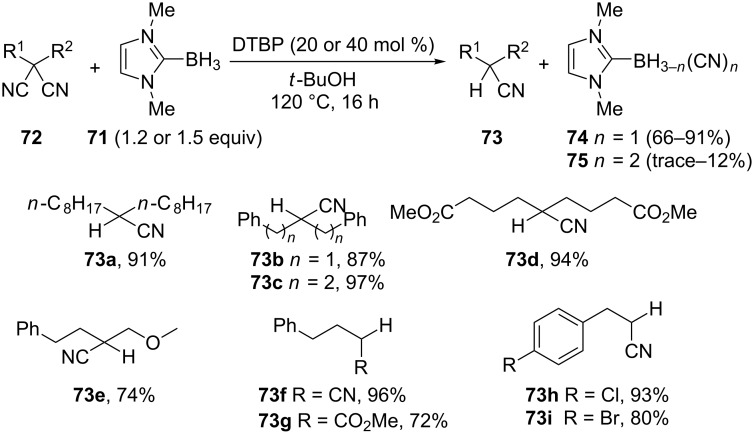
Reductive decyanation of malononitriles and cyanoacetates using NHC-boryl radicals (9 examples). For **74** and **75**, isolated or NMR yields are given from **71** [121].

Later Curran’s group discovered that NHC-boryl radicals, generated from NHC-boranes (N-heterocyclic carbene boranes), abstracted the cyano group from various organic nitriles and dinitriles and applied this reaction for the synthesis of new NHC-boryl nitrile and dinitrile compounds [121]. They observed that malononitrile was the most efficient donor to produce boryl nitriles and concluded that substituted malononitriles would be decyanated by NHC-boranes. For this transformation, malononitriles are reacting with a slight excess of NHC-borane **71**, in refluxing *t*-BuOH with DTBP (di-*tert*-butylperoxide) as radical initiator. The yields are attractive while roughly comparable amounts of boryl nitriles **74** and **75** are formed (Scheme 23). Malononitriles **72d**,**e** are successfully reduced to **73d**,**e** while with photoactivated **78** such substrates afforded complex mixtures (see below). Contrary to the reductive decyanation with Bu_3_SnH [115], the NHC borane reduces the α-cyanoester **72g** to **73g** while the aryl chloride and bromide moieties are preserved (**73h**,**i**).

**Scheme 24:**
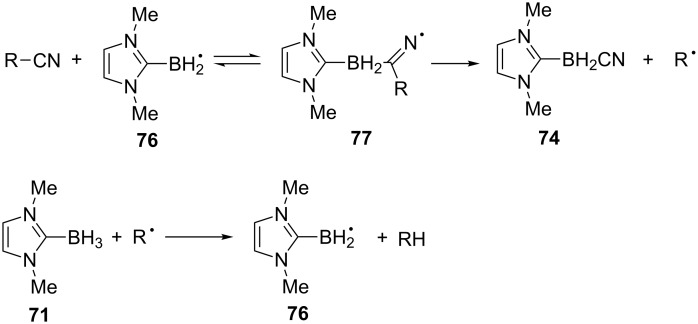
Proposed mechanism for the reduction by NHC-boryl radicals. The other possible pathway (addition of **76** to the nitrogen) is not represented here [121].

The use of a 5-hexenyl radical probe led to the formation of 99% of cyclized products (relative yield). The authors proposed a radical chain mechanism similar to that proposed in the reaction with Bu_3_SnH. While the tin radical was proposed to add to nitrogen [115], here the evidence points more to the addition of the boryl radical **76** on the nitrile carbon to form the nitrogen centered radical **77**. β-Fragmentation leads to NHC-boryl nitrile **74** and a carbon centered radical. A hydrogen atom transfer reaction between the electrophilic α-cyano radical and the nucleophile NHC-borane achieves the chain propagation (Scheme 24). The isolation of the NHC-boryl nitrile **74**, EPR spectroscopy observations [122], and polar effects fit with this proposition.

### Neutral organic electron donors

Powerful single-electron transfer reagents have been described. Kang et al. reported the decyanation of both malononitriles and α-cyanoesters using samarium(II) iodide/THF/HMPA at respectively 0 °C and room temperature [123]. Metallic samarium can also promote the decyanation [124].

**Scheme 25:**
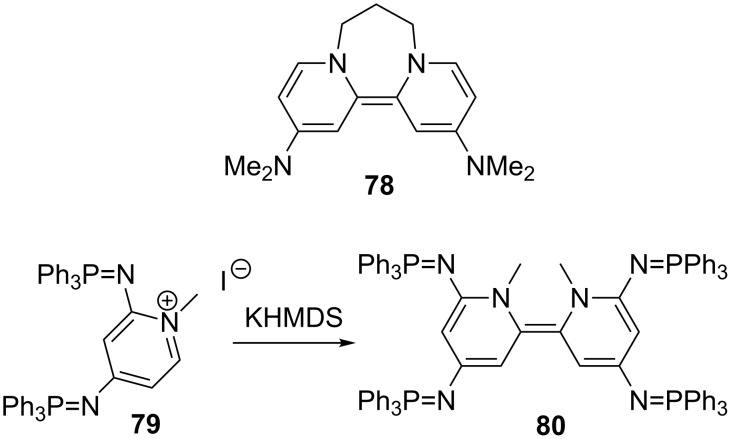
Structures of organic electron-donors. Only the major *Z* isomer of **80** is shown [125,127].

**Scheme 26:**
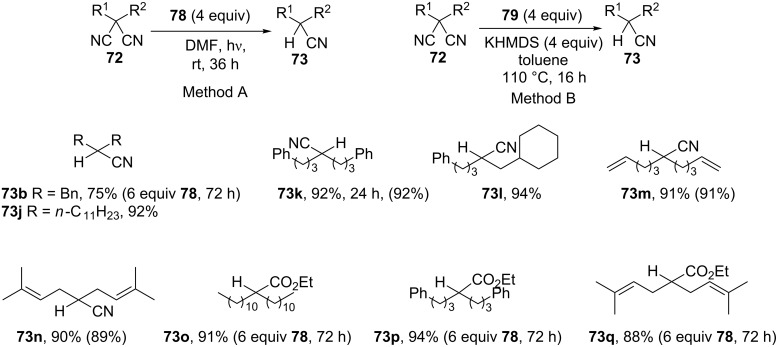
Reductive decyanation of malononitriles and cyanoacetates using organic electron-donors (method A, 11 examples; method B, 3 examples). Yields in brackets refer to method B [125,127].

Doni and Murphy have reported the reductive decyanation of malononitriles and α-cyanoesters by using the neutral organic electron donor **78** (Scheme 25) under photoactivation (method A, Scheme 26) [125]. The first observation was that surprisingly, the reaction of 2,2-dibenzylmalononitrile (**72b**) provided both debenzylated (2-benzylmalononitrile, 19%) and decyanation (**73b**, 75%) products. In contrast, the corresponding dibenzylcyanoacetate led exclusively to the debenzylation product [126]. Selected examples are presented in Scheme 26. Excellent yields are obtained even if decyanation of cyanoacetates requires higher amounts of **78** (6 equiv) with extended reaction times (72 h). In line with this work, the authors prepared the tetra(iminophosphorano)-substituted bispyridinylidene **80**, a new highly efficient neutral organic electron donor (Scheme 25) [127]. This compound is able to reduce aryl halides and appears as the only reductant able to reduce dialkylarenesulfonamides as well as malononitriles without photoexcitation. Electron donor **80** can be isolated but is more conveniently generated in situ by deprotonation with KHMDS of its pyridinium ion precursor **79** in refluxing toluene (method B).

With method B, malononitriles are reduced to mononitriles with comparable yields to that achieved with photoactivated **78** (**73k**,**m**,**n**). No allylic bond cleavage is observed and alkene moieties are preserved in the decyanated product (**73m**,**n**,**q**). No radicals are trapped from the reduction of the 5-hexenyl radical probe **72m** with both methods. Mononitrile 2,2-dimethylhexadecanenitrile appears inert with both methods, and organic bromides are not tolerated. The proposed mechanism is similar to Scheme 1. First, a SET from the electron donor to the nitrile forms a radical anion. This radical anion can fragment into a cyanide ion and a radical which is rapidly reduced into a stabilized carbanion before protonation. This carbanion appears as a key intermediate leading to complex mixtures when method A is applied to **72d** or a substrate similar to **72e**.

**Scheme 27:**
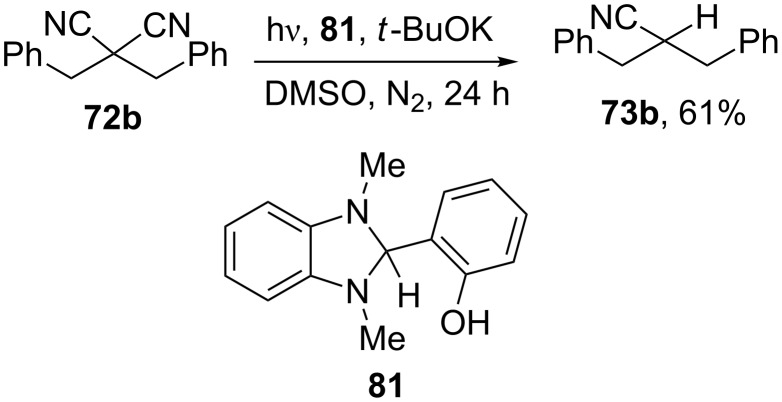
Photoreaction of dibenzylmalononitrile with **81** [128].

More recently, the reaction of dibenzylmalononitrile **72b** with the hydroxyaryl-substituted benzimidazoline derivative **81** as photo-reductant in a basic medium has been described and led to **73b** in fair yield (Scheme 27) [128].

### Acid, base or organometallic-induced reductive decyanation

**Scheme 28:**
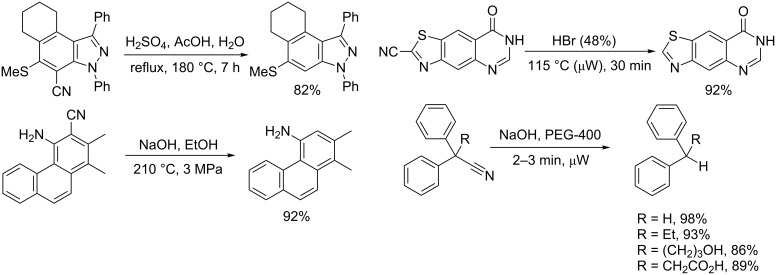
Examples of decyanation promoted in acid or basic media [129,131,134–135].

Among the other procedures mentioned in our previous review [9], the base [129–130] or acid-induced [131–134] hydrolysis–decarboxylation sequence appears as a classical pathway (Scheme 28).

**Scheme 29:**
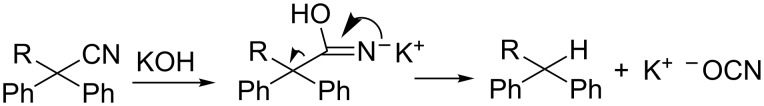
Mechanism proposed for the base-induced reductive decyanation of diphenylacetonitriles [136].

In the particular case of diphenylacetonitriles, an addition–elimination mechanism is proposed (Scheme 29) [135–136]. Such a pathway could also apply for the reductive decyanation of diphenylacetonitriles induced by organolithiums or Grignard reagents [137–138]. This reaction, applied to nitriles substituted with suitable leaving groups appears as a cyanation method of organometallic reagents or other nucleophiles [139].

**Scheme 30:**
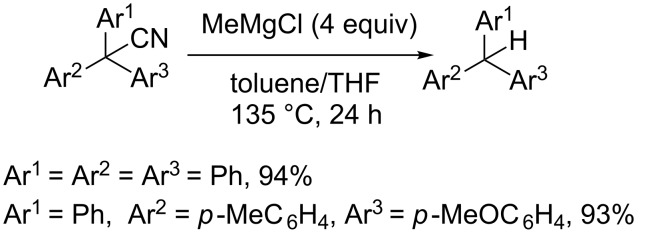
Reductive decyanation of triarylacetonitriles [140].

Nambo et al described the preparation of triarylacetonitriles using sequential Pd-catalyzed arylations. Triarylacetonitriles obtained can be transformed into various species including triarylmethanes by treatment with MeMgCl (Scheme 30) [140]. In this case a SET mechanism could operate [141].

## Conclusion

The reductive decyanation reaction appears useful in organic synthesis because it offers the possibility to temporarily use the advantages from the nitrile functional group. While our previous review showed that several methods had a narrow substrate scope [9], new and convenient synthetic methods have now emerged. Classical metal dissolving conditions are still used but the method, while it works, shows that the course of the reaction may strongly depend on reaction conditions. The transition-metal-catalyzed defunctionalization reactions cover a wide range of nitriles including the most challenging alkanenitriles and allow a large functional group tolerance. The decyanation of α-aminonitriles by aluminium- and borohydrides has been widely investigated and offers synthetic applications in heterocyclic chemistry. Recent developments using *N*-heterocyclic carbene boranes and super electron donors provide new procedures for the reduction of malononitriles or α-cyanoesters. This opens the possibility to synthetic applications by using these intermediates in the future.
